# Anterior maxilla alveolar ridge dimension and morphology measurement by cone beam computerized tomography (CBCT) for immediate implant treatment planning

**DOI:** 10.1186/s12903-015-0055-1

**Published:** 2015-06-10

**Authors:** Wenjian Zhang, Adam Skrypczak, Robin Weltman

**Affiliations:** 1 Department of Diagnostic and Biomedical Sciences, The University of Texas School of Dentistry at Houston, 7500 Cambridge Street, Houston, TX 77054 USA; 2 Junior Dental Student, The University of Texas School of Dentistry at Houston, 7500 Cambridge Street, Houston, TX 77054 USA; 3 Department of Periodontics and Dental Hygiene, The University of Texas School of Dentistry at Houston, 7500 Cambridge Street, Houston, TX 77054 USA

**Keywords:** Anterior maxilla, CBCT, Alveolar ridge, Buccal undercut, Implant

## Abstract

**Background:**

Implants have been widely used to restore missing teeth. Limited information on applied anatomy at the anterior maxilla compromises the clinical outcome for implant placement in this region. In the current study, Cone Beam Computerized Tomography (CBCT) was used to measure alveolar ridge and buccal undercut dimension at the anterior maxilla to help develop treatment planning for immediate implant placement.

**Methods:**

CBCT scans were screened to include 51 subjects with full dentition at right maxilla. Measurements were taken at the cross sectional views in the middle of the maxillary right central incisor, lateral incisor, and canine regions. Alveolar height was measured from the alveolar crest to floor of nasal fossa. Alveolar width was measured from the buccal to palatal cortical plate at the coronal, middle, and apical third of the distance from the alveolar crest to floor of the nasal fossa. Buccal undercut location was measured from where the buccal cortical plate started dipping to a line extending at the alveolar crest that was perpendicular to the long axis of the alveolar ridge. The buccal undercut depth was measured from the deepest point of the undercut at the buccal plate to a line tangent to the buccal plate paralleling the long axis of ridge.

**Results:**

Alveolar width increased from coronal to apical direction for each tooth. Mean alveolar widths (mm) were: central incisor, 9.55; lateral incisor, 8.30; canine, 9.62. The lateral incisor had a significantly smaller alveolar width than the other anterior teeth. No significant difference in ridge height was noted among the teeth. Undercut locations from the alveolar crest (mm) were: central incisor, 5.84; lateral incisor, 3.59; canine, 5.11. Undercut depths (mm) were: central incisor, 0.76; lateral incisor, 0.87; canine, 0.73. The percentages of teeth with buccal undercuts were: central incisor, 41 %, lateral incisor, 77 %, and canine 33 %. Male demonstrate significant larger ridge width compared with females for all three teeth.

**Conclusions:**

At anterior maxilla, the lateral incisor has the thinnest alveolar bone, and most frequently exhibits a buccal undercut which is the closest to alveolar ridge compared with other maxillary anterior teeth.

## Background

Oral rehabilitation with implant-supported prostheses has been very successful in restoration of single or multiple missing teeth [1–5]. Successful implant treatment depends on precise planning. Information on the height, width, morphology, and density of alveolar bone surrounding the proposed implant site is very critical for determination of the size of the implant and angle of placement [6–8].

Conventional radiographic techniques such as intraoral, panoramic, and cephalometric images used to be the standard methods for implant treatment planning [9]. However, imaging distortion and superimposition compromise the accuracy of treatment planning with these techniques [10]. The improvement in sectional imaging techniques advocates the use of tomographic technique in the investigation of potential implant sites [10]. The recent introduction of cone beam computerized tomography (CBCT) in dentistry, opens up a new horizon in providing comprehensive pre-operative implant site assessment and sophisticated surgical guide in dental implantology [11]. The American Academy of Oral and Maxillofacial Radiology (AAOMR) recently recommended CBCT as the imaging modality of choice for implant treatment planning [12].

CBCT provides high-resolution and accurate multiple planar reformatted images at a relatively low radiation dosage and affordable price [13–16]. Dimensional measurement by CBCT can achieve sub-millimeter accuracy which is comparable to the level of multi-slice computerized tomography (MSCT) [17], and precision of the measurement will not be affected by variations in voxel settings in the imaging acquisition protocol [18]. Implant length in initial planning with panoramic radiographs tends to be overestimated, which could be attributed to the inherent magnification in panoramic imaging leading to an overestimation of the available bone for implant placement [19, 20]. This inaccuracy may result in a greater risk of injury to adjacent anatomic structures, such as floor of maxillary sinus or inferior alveolar nerve. Implant sizes estimated by CBCT images are narrower and shorter than those obtained from panoramic radiographs [21], suggesting that CBCT exams lead to a safer decision.

Maxillary anterior region may be the implant site that requires the most rigorous pre-operative assessment, because alveolar dimension and morphology will have a direct influence on aesthetic outcome and stability of implant placement [22]. Previous experience has shown that adequate alveolar height is not the only prerequisite for a successful implant placement. Deficiency of transversal ridge width would lead to length reduction or even impossible implant insertion [23]. However, very few studies have evaluated bone parameters for implant treatment at this region.

In the present study, CBCT images were used to evaluate alveolar ridge dimension and the presence and size of buccal undercut at the maxillary anterior region. The correlation of ridge height and width with the age and gender of the subjects was also analyzed. This study was aimed to provide more quantitative information to help immediate implant treatment at the maxillary anterior area.

## Methods

### Subjects

The subjects who had CBCT scans performed at the University of Texas School of Dentistry at Houston Radiology Division since 2011 were screened according to the selection criteria. The exclusion criteria were: 1) systemic/endocrine diseases that influence bone metabolism, e.g., osteoporosis, hyperparathyroidism, Paget’s disease, and renal osteodystrophy; 2) topical conditions that may affect bone quantity and quality at anterior maxilla, e.g., moderate to severe periodontal disease, cyst, neoplasm, prior trauma or surgery. A total of 51 subjects with full dentition at right maxilla were included in the study. There were 20 males and 31 females, with an age range of 16–80 years old (45.25 ± 17.72, Table 1). Institutional Review Board (IRB) exemption was obtained for the study after internal review.

**Table 1:** Patient age and gender information

Age	Males	Females
16–29	5	5
30–39	3	11
40–49	2	5
50–59	4	2
60–69	3	5
70–80	3	3
Total	20	31

### CBCT imaging acquisition

All included CBCT scans covered both maxillary and mandibular arches with a field of view (FOV) of 150 x 90 mm^2^. The scans were acquired at 90 kV (kV), 10 mA (mA), 16 s, and a 0.2 mm^3^ voxel size with a Kodak 9500 unit (Carestream Health, Inc, Rochester, NY). CBCT images were reconstructed with Anatomage Invivo 5.1 software at 1 mm thickness. All images were displayed on a 19-in. flat panel screen (HP Development Company, Palo Alto, CA) with a 1920 X 1080 pixel resolution and viewed in a dimly lit environment.

### Measurements

**Fig. 1:**
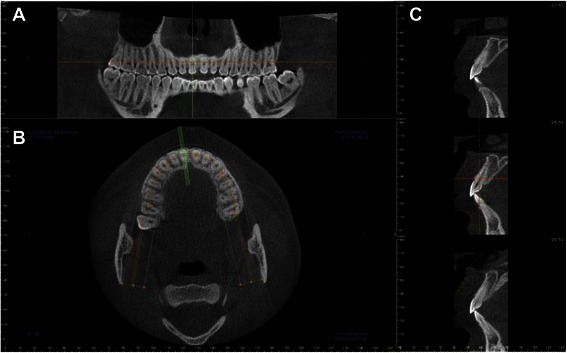
Reformatted CBCT views. **a**. Reformatted panoramic view demonstrates that the occlusal plane is parallel to the floor. **b**. Axial view at the maxillary arch level. The green lines are perpendicular to alveolar ridge. They indicate where the cross sectional views were taken. **c**. Series of cross sectional views. The view in the middle panel (corresponding to the middle green line in B) was used for alveolar volume and buccal undercut measurements

**Fig. 2:**
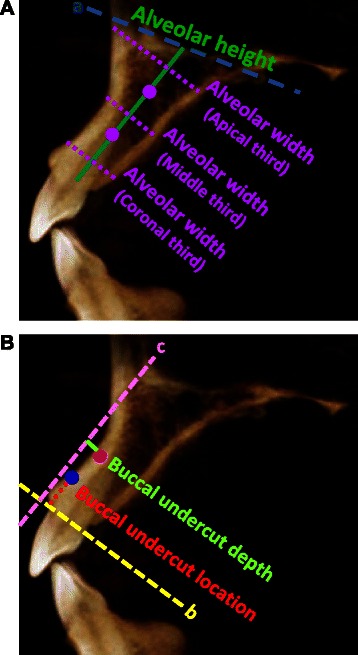
Diagrams for alveolar ridge and buccal undercut measurements. **a**. Alveolar height and width measurements. Line “a” represents the floor of nasal fossa. Green line represents the distance from alveolar crest to the floor of nasal fossa, and is designated as alveolar height. The alveolar height is divided into thirds (shown by the purple dots). In the middle of each third, a dotted purple line is drawn perpendicular to the long axis of the ridge and extends from buccal to palatal cortical plate. The distance between the two plates is designated as alveolar width at apical third, middle third, and coronal third, respectively. **b**. Buccal undercut location and depth measurements. Line “b” is the alveolar crest line perpendicular to the long axis of alveolar ridge. Blue dot is where buccal cortical plate starts dipping. The distance from the blue dot to line “b” is designated as buccal undercut location. Line “c” is tangent to buccal cortical plate and parallel to the long axis of alveolar ridge. The pink dot represents the deepest point on the buccal undercut. The green line representing the distance between the deepest point and tangent line is designated as the buccal undercut depth

**Fig. 3:**
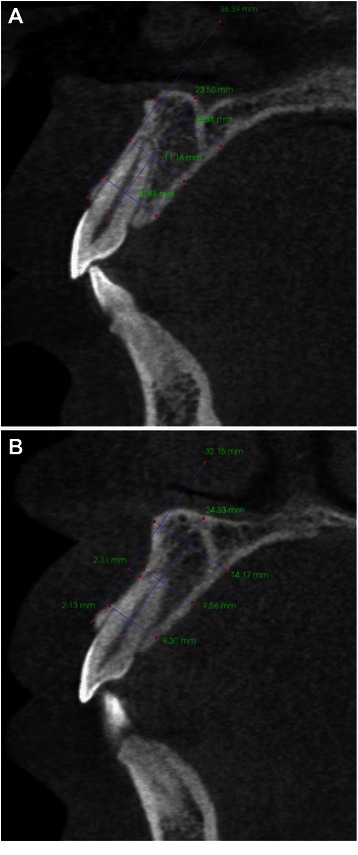
Cross sectional views demonstrate absence or presence of buccal undercut. **a**. No buccal undercut for this maxillary right canine. **b**. Presence of buccal undercut for this maxillary right lateral incisor

To ensure consistent head placement, all CBCT scans were checked and re-orientated, if necessary, to position the occlusal plane parallel to the floor (Fig. 1a). Cross sectional views perpendicular to alveolar ridge were taken in the middle of maxillary right central incisor, lateral incisor, and canine regions (Fig. 1b and c). The linear measurements were done as described below (also see Fig. 2). Absence or presence of buccal undercut was demonstrated in a right maxillary canine (Fig. 3a) and a right maxillary lateral incisor (Fig. 3b), respectively. All the measurements were taken by one examiner.

1. Alveolar height
  A line was drawn from alveolar crest paralleling with the long axis of alveolar ridge. The distance from alveolar crest to the floor of nasal fossa was defined as alveolar height. (Fig. 2a).
2. Alveolar width
  Alveolar height was divided into coronal, middle, and apical third. In the middle of each third, a line was drawn perpendicular to the long axis of alveolar ridge. The distance between buccal and palatal cortical plate was defined as alveolar width (Fig. 2a). The overall alveolar width for each tooth was the average of coronal, middle, and apical third of alveolar width measurements.
3. Buccal undercut location
  For a tooth identified to have buccal undercut, a line was extended from alveolar crest which was perpendicular to the long axis of the alveolar ridge. The distance from where the buccal cortical plate started dipping to the aforementioned line was defined as buccal undercut location (Fig. 2b). This value demonstrated how close the buccal undercut was to the alveolar crest.
4. Buccal undercut depth
  For a tooth identified to have buccal undercut, a line tangent to buccal cortical plate and parallel to the long axis of alveolar ridge was drawn. The distance from the deepest point of the buccal undercut to the aforementioned line was defined as the buccal undercut depth (Fig. 2b).
5. Percent of teeth with buccal undercut
  For maxillary right central incisors, lateral incisors, and canines, the formula to calculate the percent of teeth with buccal undercut was: (the number of teeth with buccal undercut)/(total number of teeth evaluated)X100.

### Statistical analysis

Kolmogorov–Smirnov test was used to determine the normality of the data. One-way ANOVA followed by Tukey's honestly significant difference (HSD) test and Kruskal-Wallis test were used to detect statistical difference among the groups for normal and non-normal distributed data, respectively. The correlations between subjects’ age and gender with alveolar height and width measurements were evaluated by Spearman’s correlation analysis. Data were reported as means ± standard deviation (SD). The statistical difference was set at a p value less than 0.05. All of the statistical analysis was run with SAS 9.2 program (SAS Institute Inc., Cary, NC).

## Results

The normality of the data was tested by Kolmogorov–Smirnov test. It was found that the data for alveolar height and width had normal distribution, therefore, one-way ANOVA followed by Tukey's honestly significant difference (HSD) test was used to detect statistical difference among the three maxillary anterior teeth. The data for buccal undercut location and depth had non-normal distribution, and Kruskal-Wallis test was used to detect the statistical difference among the teeth.

**Fig. 4:**
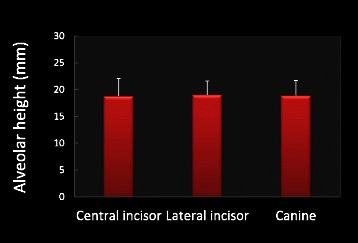
Alveolar height measurements. There is no significant difference in alveolar height among maxillary central incisor, later incisor, and canine. *N* = 51 for all three teeth

**Fig. 5:**
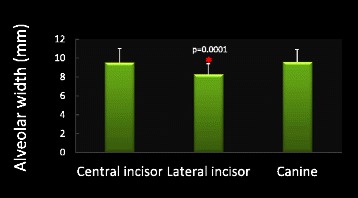
Alveolar width measurements. Lateral incisor demonstrates significantly thinner alveolar ridge compared with central incisor and canine. Asterisk denotes *p* = 0.0001. *N* = 51 for all three teeth

**Fig. 6:**
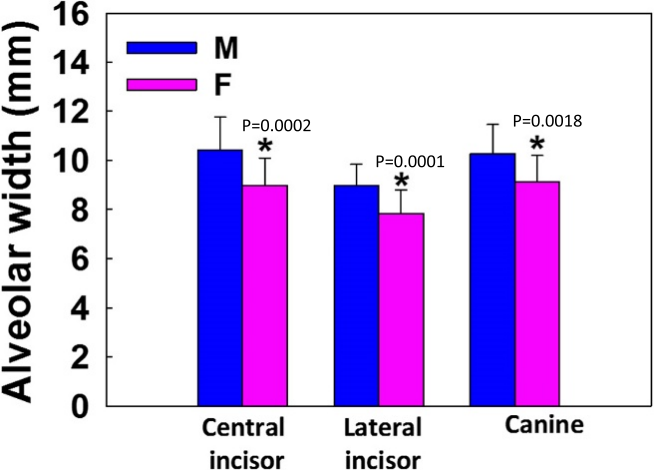
Male demonstrates significant larger alveolar width compared to female for maxillary central incisor, lateral incisor, and canine. Stars indicate statistically significant difference (*p* = 0.0002, 0.0001, and 0.0018 for central incisor, lateral incisor, and canine, respectively). *N* = 20 for male, *N* = 31 for female for all three teeth

Mean alveolar height for the maxillary right central incisor, lateral incisor, and canine was 18.83 ± 3.23, 19.07 ± 2.53, 18.91 ± 2.81 mm, respectively. There was no significant difference in the ridge height among these teeth (Fig. 4). Coronal, middle, and apical third alveolar width for maxillary right central incisors was 8.07 ± 0.93, 8.67 ± 1.62,11.91 ± 2.38 mm, lateral incisors was 7.08 ± 0.80, 7.35 ± 1.39, 10.48 ± 1.81 mm, and canines was 8.94 ± 1.08, 8.72 ± 1.35, 11.19 ± 2.06 mm, respectively. The alveolar width increased from the coronal to apical direction for all three teeth. The mean alveolar width for maxillary central incisors, lateral incisors, and canines was 9.55 ± 1.45, 8.30 ± 1.10, 9.62 ± 1.30 mm, respectively. The lateral incisors demonstrated significantly thinner alveolar width than the other two anterior teeth (*p* = 0.0001, Fig. 5). For the correlation between subjects’ age/gender and alveolar volume measurements, it was found that male demonstrated significantly larger alveolar width compared to female for all three maxillary anterior teeth (Fig. 6). Male and female alveolar width (mm) for maxillary right central incisor were 10.41 ± 1.36 and 8.96 ± 1.14, (*r* = 0.5, *p* = 0.0002), for maxillary right lateral incisor were 8.97 ± 0.87, 7.84 ± 0.94 (*r* = 0.52, *p* = 0.0001), for maxillary right canine were 10.26 ± 1.20 and 9.13 ± 1.07 (*r* = 0.44, *p* = 0.0018), respectively (Fig. 6).

**Fig. 7:**
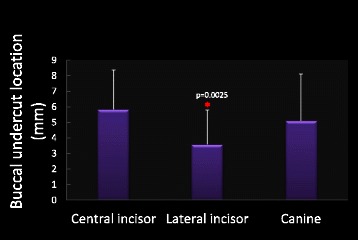
Measurements for buccal undercut location. Lateral incisor has buccal undercut which is the closest to alveolar ridge compared with central incisor and canine. Asterisk denotes *p* = 0.0025. *N* = 51 for all three teeth

**Fig. 8:**
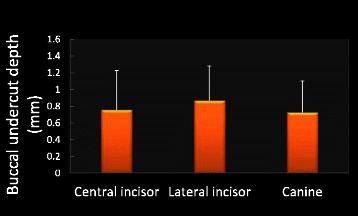
Measurements for buccal undercut depth. There is no significant difference in buccal undercut depth among maxillary central incisor, later incisor, and canine. *N* = 51 for all three teeth

Among the maxillary right anterior teeth, 41 % of central incisors, 77 % of lateral incisors, and 33 % of canines had buccal undercut. The mean distance of buccal undercuts to the alveolar ridge (mm) for central incisors, lateral incisors, and canines was 5.84 ± 2.52, 3.59 ± 2.21, 5.11 ± 2.99, respectively. The buccal undercut for lateral incisors was the closest to alveolar ridge compared to the other anterior teeth (*p* = 0.0025, Fig. 7). The buccal undercut depth (mm) for central incisor, lateral incisor, and canine was 0.76 ± 0.47, 0.87 ± 0.41, 0.73 ± 0.37, respectively. There was no statistically significant difference in buccal undercut depth among these three maxillary anterior teeth (Fig. 8).

## Discussion

The alveolar process after tooth extraction normally undergoes resorption resulting in decreased alveolar height and width [24–28]. The alveolar dimension prior to tooth extraction is considered one of the prognostic factors in determining the available alveolar volume for implant placement following extraction [29]. It has been a general consensus that a precise preoperative evaluation of alveolar dimension at the future implant site is very important to develop an appropriate placement strategy and to preserve adjacent anatomical structures, especially for cases in need of immediate implant placement [23]. In the anterior maxilla, implant placement presents more challenges due to the demand for well-anchored implant as well as for satisfactory esthetic result [30, 31].

There is scarce information in the current literature on the alveolar dimension in the maxillary anterior area. Several studies have evaluated the buccal bone wall thickness at anterior maxilla, and the data suggest that a minimal 2 mm in thickness is ideal to achieve an optimal biological and esthetic outcome [32–34]. However, the overall alveolar dimension and morphology at anterior maxilla have not been fully evaluated yet. In the present study, the averaged alveolar height and width at maxillary anterior region ranged between 18.83 ~ 19.07 mm and 8.30 ~ 9.62 mm, respectively, for the selected population.

The lateral incisor had the thinnest alveolar ridge compared with the central incisor and canine, probably due to the presence of a lateral fossa which creates the buccal concavity adjacent to lateral incisor [35]. The alveolar width increased from the coronal to apical direction for all three anterior teeth, demonstrating a general bell curve-shaped ridge in anterior maxilla. Male demonstrated significantly wider alveolar ridge compared to female for all three maxillary anterior teeth, which was consistent with what was reported in the literature for other dentoalveolar region [29]. All the subjects included in the study had full dentition at right maxillary anterior region, which eliminated the influence of alveolar atrophy due to edentulism.

In the current study, 41 % of central incisors, 77 % of lateral incisors, and 33 % of canines have been found to have buccal undercuts. Except for the much higher incidence of undercut associated with lateral incisor, this result was similar to what has been reported for mandibular posterior area [29, 36]. Presence of lingual undercuts above the mandible canal was observed in 36-39 % of mandible molars [29, 36]. A buccal or lingual undercut increases the risk of alveolar cortical plate perforation and surgical complication, or indicates the need for additional grafting procedures. To compensate for this anatomical variation, an implant may have to be placed off-axially and restored with an angled abutment [37].

Based on the current study, it appears that without additional grafting procedures, implant placement in the lateral incisor region would incur highest risk of perforation of the buccal plate, whereas the canine region would be the least likely for such an event in the anterior maxilla. The lateral incisor has the thinnest alveolar ridge and highest incidence of buccal undercut. In addition, its undercut is most coronally positioned among the three anterior teeth. The parameters for canine were opposite for the most part. A careful preoperative evaluation of anterior maxilla, especially of the lateral incisor region, is invaluable for selection of the optimal treatment approach and reducing surgical complications.

Although we minimized the variables as much as possible, there are still some limitations in the study. Some of these include a relatively small sample size and variations in ethnicities of patients. Future investigation with larger sample size and different ethnic background would be needed to further validate current findings.

## Conclusions

An average alveolar dimension at anterior maxilla is approximately 18 ~ 19 mm in height and 8 ~ 9 mm in width for the selected population. At least one third of maxillary anterior teeth have buccal undercut with various depth and location. Careful treatment planning with CBCT is critical for successful implant placement, especially at the lateral incisor region due to limited availability of alveolar bone.

## Abbreviations

CBCT: Cone Beam Computerized Tomography
AAOMR: The American Academy of Oral and Maxillofacial Radiology
MSCT: Multi-slice Computerized Tomography
IRB: Institutional Review Board
FOV: Field of View
KV: Kilovolt
MA: Milliampere
HSD: Tukey's honestly significant difference
SD: Standard Deviation
